# Chloride salt enhances plant resistance to biotic stresses

**DOI:** 10.3389/fpls.2024.1385164

**Published:** 2024-06-04

**Authors:** Yu-Bing Yang, Chang Yang, Jia-Rui Zheng, Liang-Zheng Xu, Nan Yao

**Affiliations:** ^1^ School of Life Sciences, Jiaying University, Meizhou, Guangdong, China; ^2^ State Key Laboratory of Biocontrol and Guangdong Provincial Key Laboratory of Plant Resources, School of Life Sciences, Sun Yat-sen University, Guangzhou, China

**Keywords:** salt stress, cell death, broad spectrum resistance, salicylic acid, Arabidopsis

## Abstract

Biotic stresses caused by bacterial and fungal pathogens damage crops; identifying treatments that enhance disease resistance provides important information for understanding plant defenses and sustainable agriculture. Salt stress affects crop yields worldwide; however, studies have focused on the toxic sodium ion, leaving the effects of the chloride ion unclear. In this study, we found that irrigation with a combination of chloride salts (MgCl_2_, CaCl_2_, and KCl) suppressed the cell death phenotype of the ceramide kinase mutant *acd5*. Chloride salt pre-irrigation also significantly limited the cell death caused by *Pseudomonas syringae* pv *maculicola* infection and inhibited the multiplication of this bacterial pathogen in a mechanism partially dependent on the salicylic acid pathway. Moreover, chloride salt pre-irrigation improved plant defenses against the fungal pathogen challenge, confining the lesion area caused by *Botrytis cinerea* infection. Furthermore, the growth of herbivorous larvae of *Spodoptera exigua* was retarded by feeding on chloride salt irrigated plants. Thus, our data suggest that treatment with Cl^-^ increases broad spectrum resistance to biotic challenges.

## Introduction

Plants have evolved many ways to defend against abiotic and biotic stresses. Different stressors provoke different defense response pathways and affect plant responses to subsequent stressors via synergistic or antagonistic crosstalk. Since the toxic threshold for Na^+^ is much lower than that of Cl^-^, researchers have focused on the mechanisms that respond to Na^+^ stress, rather than the effect of Cl^-^ on plants. Under high-salt conditions, plants exclude ions such as Na^+^ from root cells to reduce uptake or unload ions from the xylem to the phloem to decrease ion transport to the shoot (Davenport et al., 2007; Zhu, 2016). The accumulation of osmotic substances helps to balance water potential and inhibit the influx of toxic ions from the apoplast into the cytosol (Hasegawa et al., 2000). In addition, plant cells compartmentalize high levels of Na^+^ in vacuoles to keep ion concentrations below the toxic level in the cytosol and other organelles; some plants can also secrete ions through salt glands (Munns and Tester, 2008).

Cl^-^ accumulation can also dampen plant growth and reducing Cl^-^ levels increases plant resistance to salinity; however, several studies have demonstrated that a suitable concentration of Cl^-^ is vital for plant cell biology. For example, Cl^-^ working as an osmotic regulator affects stomata behavior and participates in plant responses to drought and salinity (Wang et al., 2013; Geilfus et al., 2015; Zhang et al., 2017). Increasing Cl^-^ contents in plants by treating the plants with Cl^-^ salts can enhance the osmotic regulatory function of NO_3_^-^ and increase the usage of NO_3_^-^ (Wege et al., 2017; Rosales et al., 2020). Franco-Navarro et al. (2019) showed that Cl^-^ can promote water use efficiency and CO_2_ diffusion in the intercellular space, thus increasing photosynthetic efficiency. Taken together, these observations suggest that a certain concentration of Cl^-^ plays a positive role in plant resistance to abiotic stress and promotes plant growth.

In addition to its effect on abiotic stress, some studies suggest that Cl^-^ affects biotic stress responses. For example, the chloride channel family protein CLCd can negatively regulate microbe pattern-triggered immunity (PTI), possibly via altered pH in the Golgi complex (Guo et al., 2014b). Pharmacological treatments showed that anion channels differentially participated in PTI and effector-triggered immunity (Guo et al., 2014a), but whether changing Cl^-^ levels by irrigation would affect plant biotic responses remains unclear.

Yang et al. (2019) found that pre-irrigation with 300 mM NaCl drastically inhibited the cell-death phenotype of *acd5* mutants, which lack ceramide kinase and exhibit accelerated cell death (Liang et al., 2003). To further investigate the molecular mechanism of the inhibitory effect of salt on cell death in *acd5*, we tried several kinds of salts to discover the main ions that affect cell death and found that Cl^-^ is the major ion suppressing cell death in *acd5*. Cl^-^ pre-irrigation also enhanced plant resistance to a broad range of biotic stresses, including hemibiotrophic and necrotrophic pathogen infection, and phytophagous insect attack. These results showed that treatment with a certain concentration of chloride salts enhanced plant resistance to a broad spectrum of biotic stresses. Further research to decipher the molecular relationships between Cl^-^ and plant cell death, plant responses to biotic stresses, will deepen our understanding of the functions of inorganic ions in cellular processes. Our data provide new insight into the biological significance of Cl^-^ as beneficial ion.

## Materials and methods

### Plant material and growth conditions

Arabidopsis wild-type (Col-0) and *acd5* mutant plants were used in this study. Seeds were surface sterilized with 70% ethanol for 7 min, washed three times with sterile water, and sown on half-strength Murashige and Skoog (MS) medium plates supplemented with 1% sucrose and 0.6% agar. After 2 days of stratification at 4°C, plates were kept in the greenhouse at 22°C and a 16-h light/8-h dark cycle for 8 days. Then the seedlings were transplanted onto soil and grown in the same greenhouse.

### Salt treatment

Three-week-old soil-grown plants were irrigated with water, 300 mM chloride salt solution (composed of 60 mM MgCl_2_, 60 mM CaCl_2_, and 60 mM KCl), or other salt solutions as specified in the figure legends once to soil capacity, typically, 15 mL of salt solution was irrigated into one well of the 32-well seedling-planting tray. Thereafter, the plants were irrigated with water as required (Yang et al., 2019). For salt spraying treatment, chloride salt solutions of different concentrations containing 0.02% Silwet-77 were sprayed onto plants. The phenotype of plants was recorded as indicated in each figure legend.

### Pathogen infection and insect feeding assay

Bacterial infection was conducted as previously described (Yang et al., 2019). Briefly, after 24 h of Cl^-^ treatment, the 3^rd^ and 4^th^ leaves of three-week–old plants were infiltrated with 10 mM MgSO_4_ (mock), *Pseudomonas syringae* pv *maculicola* (*Pma*) DG3, DG6, or *Pseudomonas syringae* pv *tomato* (*Pst*) DC3000-GFP at OD_600_ as described in the corresponding figure legend. The virulent *Pma* DG3 is a *recA* derivative of *Pma* ES4326, and the avirulent *Pma* DG6 is a *recA* derivative of *Pma* ES4326 expressing the type-III effector *avrRpt2* (Yao and Greenberg, 2006). These two pathogens have been maintained in Dr. Yao’s lab for years after being gifted by Dr. Greenberg. For the bacterial growth assay, leaf discs were collected at indicated time points to determine the population of bacteria as colony forming units. The colonization of DC3000-GFP was visualized under a fluorescence microscope (DM5000B, Leica).

*Botrytis cinerea* infection was performed as previously described (Zeng et al., 2021) with a minor modification. *B. cinerea* was cultured on V8 medium according to Zhang et al. (2015b) for 7 days. *B. cinerea* spores were suspended in potato dextrose broth (PDB) and adjusted to 1 × 10^5^ spores mL^-1^. Five μL spore suspension was dropped onto the 3^rd^ and 4^th^ leaf of the plants. Then plants were covered with a dome and kept in the dark. The *Spodoptera exigua* larvae (2nd instar) were purchased from Henan Jiyuan Baiyun Industry (China). The feeding assay was performed as previously described (Huang et al., 2021).

### Detection of flg22-induced ROS

Detection of the ROS induced by flg22 was performed according to Yang et al. (2019). Leaf discs were incubated with distilled water overnight in the dark. Then distilled water was substituted with 34 μg/mL L-012 (Wako), 20 μg/mL peroxidase, and 100 nM Flg22 or a Mock treatment without Flg22. Flg22-induced ROS was detected with a microplate detector (Spark, Tecan) by recording the relative light units (RLU) of luminescence.

### Gene expression analysis by RT-qPCR

The expression of *FRK1* (At2g19190) was detected as described previously (Yang et al., 2019). Three hours post Flg22 infiltration, the samples for total RNA isolation were collected. Total RNA was extracted using the E.Z.N.A. plant RNA Kit (R6827-01, Omega) according to the manufacturer’s instructions. One µg RNA was reverse-transcribed into cDNA using the PrimeScript RT kit with genomic DNA Eraser (Takara). Ten µL reaction system containing 1 µL of diluted cDNA sample (1:20), 5 µL SYBR Premix Ex Taq II (Takara), and 0.4 µL gene-specific primers was mixed in each 96-well plate well and then quantitatively analyzed by quantitative PCR (Step One plus, ABI). *ACTIN2* (At3g18780) was analyzed as the internal reference gene to normalize values for transcript abundance. The 2^-△△CT^ method (Livak and Schmittgen, 2001) was used to calculate the relative expression level of *FRK1*. Three technical replicates were performed for each template and primer combination. The primers for amplification are as follows: *FRK1*-F, 5′-GCCAACGGAGACATTAGAG-3′; *FRK1*-R, 5′-CCATAACGACCTGACTCATC-3′; *ACTIN2*-F, 5′-GGTAACATTGTGCTCAGTGGTGG-3′; *ACTIN2*-R, 5′-GGTGCAACGACCTTAATCTTCAT-3′.

### Transcriptome sequencing

Transcriptome sequencing was conducted on the Illumina PE150 platform by Verygenome Technology (Guangzhou, China). The clean reads were aligned to the reference genome using HISAT2 (Sirén et al., 2014). Differential expression analysis was performed using the ‘DESeq2’ R package (1.18.0) (Wang et al., 2010). Differentially expressed genes were defined by log_2_(fold change) ≥ 1 and false discovery rate (FDR) < 0.05. Gene Ontology analysis was performed using the clusterProfiler R package (Yu et al., 2012) with FDR < 0.05. We have deposited the full RNA sequencing data in NCBI (PRJNA1073023, https://www.ncbi.nlm.nih.gov/sra/PRJNA1073023).

### Histochemical staining

Cell death was visualized by trypan blue staining as previously described (Yang et al., 2019; Zeng et al., 2021). Plant leaves collected at the indicated time points were submerged in staining buffer (lactic acid: water-saturated phenol: glycerol: distilled water = 1:1:1:1, V:V:V:V) containing 0.25 mg/mL trypan blue, and incubated in boiling water for 2 min, then decolorized in chloral hydrate solution. The blue cell death lesions were recorded by a stereo microscope with the ×20 objective (SteREO Lumar.V12, Carl Zeiss), and analyzed by ImageJ software (ImageJ 1.48v).

Callose deposition was detected by aniline blue staining (Bi et al., 2014). At 12 hours post infiltration with 100 nM Flg22 or Mock (10 mM MgSO_4_), leaf samples were fixed in FAA (formaldehyde: acetic acid: ethanol: water=2:1:9:8, V:V:V:V) for 5 min. Then the samples were transferred into alcoholic lactophenol (95% ethanol:lactophenol=2:1, V:V), and incubated in boiling water for 2 min. The leaves were washed twice in 50% ethanol for 2 min. After being rinsed in distilled water, the samples were transferred into aniline blue staining buffer (0.01% aniline blue in 150 mM K_2_HPO_4_, pH 9.5) overnight, and visualized under a fluorescence microscope with the ×5 objective (DM5000B, Leica) and analyzed by ImageJ software.

### Statistical analysis

Data are presented as means ± SE. Significant differences were determined by ANOVA *post hoc* tests (*P* < 0.05) using different letters or determined by Student’s *t*-test with *P*-values presented in each figure legend. Numbers of biological replicates or technical repeats are also given in each figure legend.

## Results

### Cl^-^ treatment suppresses cell death in the *acd5* mutant

To decipher the mechanism by which 300 mM NaCl inhibits cell death in *acd5* mutants, we tested salts with different anions or cations. As shown in **Supplementary Figure S1A**, the cell death lesions on *acd5* were thoroughly suppressed by irrigation with 300 mM NaCl, 300 mM KCl, and 150 mM CaCl_2_, but were not suppressed by 300 mM NaNO_3_, 300 mM KNO_3_, 150 mM Na_2_SO_4_, or 150 mM K_2_SO_4_. This result suggests that the major ion suppressing cell death in *acd5* is chloride (Cl^-^).

Furthermore, we tested a compound salt containing MgCl_2_, CaCl_2_, and KCl (Zhang et al., 2015a) to confirm the effect of Cl^-^ on cell death. Indeed, 300 mM Cl^-^ (60 mM MgCl_2_, 60 mM CaCl_2_, 60 mM KCl) also suppressed cell death in *acd5* (**Supplementary Figure S1B**). Together, these results show that a certain concentration of Cl^-^ can suppress the cell death caused by interrupted sphingolipid metabolism. To limit the influence of individual cations, we used the MgCl_2_–CaCl_2_–KCl compound solution for Cl^-^ treatment in subsequent experiments described below.

### Cl^-^ treatment suppresses the cell-death induced by pathogens

**Figure 1:**
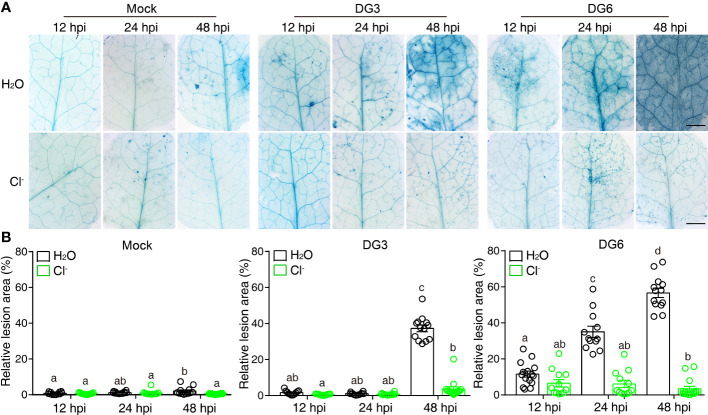
Cl^-^ treatment hinders cell death lesions induced by bacterial pathogen. Three-week-old soil-grown plants were irrigated with H_2_O or 300 mM Cl^-^. 24 h later, the 3^rd^ and the 4^th^ leaf were infiltrated with Mock (10 mM MgSO_4_), *Pseudomonas syringae* pv *maculicola* (*Pma*) DG3 (OD = 0.001), or *Pma* DG6 (OD = 0.001). **(A)** Representative phenotype of cell death visualized by trypan blue straining. Samples were detached and stained at the indicated time points, bar = 2 mm. **(B)** Statistical analysis of cell-death lesions in **(A)**. The relative cell death lesion area per leaf was quantified lesion area as a percentage of the leaf area in leaves photographed under a stereomicroscope and measured by Image J software (n ≥ 10). Data are presented as mean values ± SEM. Statistical differences were analyzed by ANOVA *post hoc* tests (*P* < 0.05) and significantly different samples are indicated with different letters. This experiment was repeated at least twice with similar results using independent samples.

Since NaCl also suppresses cell death lesions caused by pathogenic bacteria and enhances plant resistance (Singh et al., 2014; Yang et al., 2019), we next tested whether Cl^-^ could recapitulate this effect. To this end, plants pretreated with Cl^-^ were challenged with virulent *Pseudomonas syringae* pv. *maculicola* (*Pma*) DG3 and avirulent *Pma* DG6 (carrying the effector protein avrRpt2). *Pma* DG3 produces cell death induced by infection and *Pma* DG6 produces cell death as part of the hypersensitive response and disease resistance. As shown **Figure 1**, Cl^-^ irrigation suppressed the cell death induced by *Pma* DG3 and *Pma* DG6, indicating Cl^-^ could suppress cell death that resulted from susceptibility in response to virulent pathogen infection, and to resistance (hypersensitive response-related cell-death) in response to avirulent pathogen infection. We also tested whether the cations introduced by the chloride salt altered plant response to pathogenic bacteria. As shown in **Supplementary Figure S2**, although Cl^-^ salt could effectively suppress the cell death induced by *Pma* DG3, the solution containing equivalent cations, such as 60 mM K^+^, 60 mM Mg^2+^, and 60 mM Ca^2+^ released by KNO_3_, 60 mM MgSO_4_, and 60 mM Ca(NO_3_)_2_, respectively, was ineffective to confine the cell death caused by DG3 infection. This result further confirmed that chloride ion irrigation could substantially alter plant cell fate confronting pathogen challenge. Moreover, spraying with Cl^-^ suppressed the lesion development caused by spray infection with DG3 (**Supplementaryray Figure S3**). Thus, these results suggested that Cl^-^ treatment could not only suppress the cell death in the lesion mimic mutant *acd5*, but could also suppress cell death induced by bacterial pathogens.

**Figure 2:**
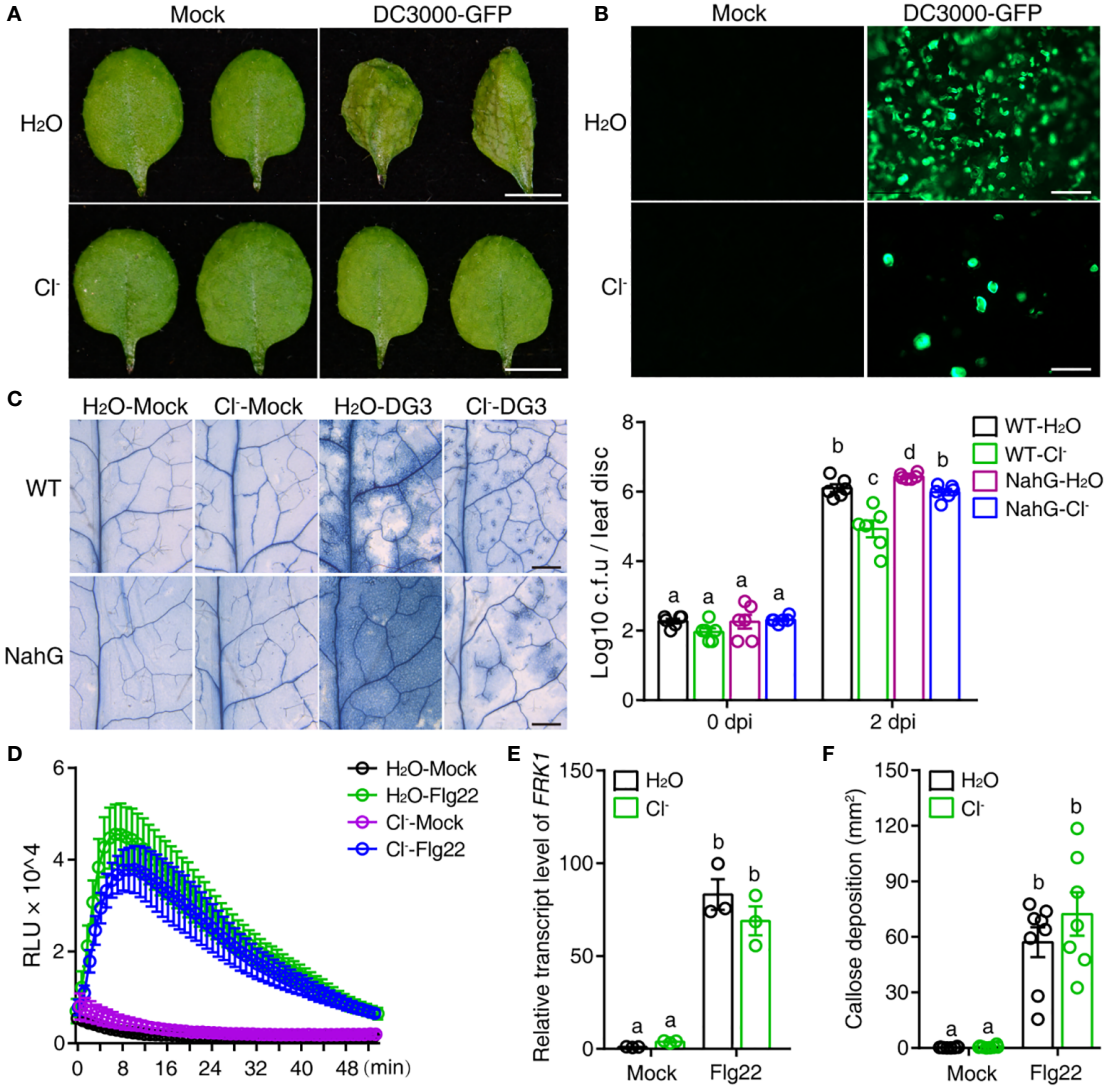
Cl^-^ treatment increases plant resistance to bacterial pathogens. Three-week-old soil-grown plants were irrigated with H_2_O or 300 mM Cl^-^. 24 h later, the 3^rd^ and the 4^th^ leaves were infiltrated with Mock (10 mM MgSO_4_), or pathogenic agents, specifically DG3 (OD = 0.0005) or *Pseudomonas syringae* pv *tomato* (*Pst*) DC3000-GFP (OD = 0.01). **(A)** Representative phenotypes of leaves recorded 2 dpi with Mock or *Pst* DC3000-GFP infiltration, bar = 5 mm. **(B)** The proliferation of DC3000-GFP in leaves observed by fluorescence microscopy at 2 dpi, bar = 100 μm. **(C)** Representative phenotype of cell death visualized by trypan blue straining at 2 days post DG3 infection (dpi), bar = 1 mm. DG3 growth in inoculated leaves shown in the right panel. Leaf discs were taken at the indicated time (n = 6). **(D)** The reactive oxygen species (ROS) burst induced with 100 nM Flg22 or without Flg22 (Mock). At 24 h after chloride salt irrigation, leaf discs from the 3^rd^ and 4^th^ leaves were collected and prepared for Flg22-induced ROS detection (for Flg22 induction, n = 5; for Mock, n = 3). **(E)** Relative transcript level of *FRK1* (At2g19190) upon Flg22 induction. RNA samples were collected 3 h post infiltration with 100 nM Flg22. **(F)** Quantification of callose deposition upon Flg22 or Mock (10 mM MgSO_4_) induction post salt treatment. Leaves were collected 12 h post infiltration and stained with aniline blue (n ≥ 7). Callose deposition per visual field was counted via Image J software. Data are presented as mean values ± SEM. Statistical differences were analyzed by ANOVA *post hoc* tests (*P* < 0.05) and significantly different samples are indicated with different letters. These experiments were repeated at least twice using independent samples.

Plant cell death is tightly related to resistance to pathogens. Therefore, we analyzed the interactions between plants and different pathogens to decipher the impact of Cl^-^ treatment on pathogenesis. GFP-tagged *Pseudomonas syringae* pv. *tomato* (*Pst*) DC3000 (DC3000-GFP) was inoculated to visualize *Pst* colonization of the phyllosphere *in situ* (Kong et al., 2020). As shown in **Figures 2A, B**, DC3000-GFP remained pathogenic to Arabidopsis, and multiplied in plants irrigated with H_2_O, but not in plants irrigated with Cl^-^.

Salicylic acid (SA) is the major phytohormone supporting plant resistance to bacterial pathogens. The Arabidopsis transgenic line NahG, which overexpresses a bacterial SA hydroxylase, accumulates little or no SA, leading to plant susceptibility to pathogens. In the present study, consistent with previous reports, NahG increased plant susceptibility to DG3, as indicated by much more severe cell death and more robust bacterial proliferation compared with control plants (**Figure 2C**). Intriguingly, Cl^-^ pretreatment also suppressed the cell death induced by DG3 and dampened bacterial growth in NahG plants (**Figure 2C**). However, the multiplication of DG3 in NahG-Cl^-^ was still significantly higher than that in WT-Cl^-^ treatment, indicating that the SA pathway played a role in Cl^–^mediated enhancement of plant resistance to DG3.

Previously, Singh et al. (2014) discovered that certain abiotic stresses prime PTI, thus enhancing plant resistance to a subsequent biotic challenge. To check whether Cl^-^ treatment also changed the properties of PTI, we examined responses to a molecular pattern, the Flg22 epitope of flagellin. As shown in **Figure 2D**, Flg22 induced a rapid reactive oxygen species (ROS) burst in plants treated with Cl^-^ and control plants treated with H_2_O. Cl^-^ treatment did not significantly alter the relative expression level of the PTI marker gene *FRK1* and Flg22-induced callose deposition (**Figures 2E, F**). These results suggested that, in the present study, the enhancement of plant resistance to bacterial infection was partially dependent on the SA pathway, but not PTI responses.

To decipher the effect of Cl^-^ treatment on plant resistance, we next conducted transcriptome analysis. To this end, samples collected 9 hours post bacterial inoculation (hpi) were sequenced and analyzed (**Supplementary Figure S4**). Compared with H_2_O-Mock, C1^–^Mock treatment downregulated biotic resistance–related genes (**Supplementary Figures S4B, D**). This is consistent with a report that salt treatment can suppress the SA pathway (Yang et al., 2019). Consistent with the observed course of DG3 infection, the genes related to immune response, such as SA, jasmonate, and ethylene pathways, were strongly induced in plants infected with DG3 after H_2_O and Cl^-^ pretreatment compared to H_2_O-Mock, Cl^–^Mock, respectively (**Supplementary Figures S4B, D**). Genes related to response to toxic substance and benzene-containing compound metabolic process were upregulated in Cl^–^DG3 compared with H_2_O-DG3, suggesting these two processes might take part in the mechanism by which Cl^-^ enhances plant disease resistance.

### Cl^-^ treatment enhances plant resistance to pathogenic fungi and an herbivorous insect

**Figure 3:**
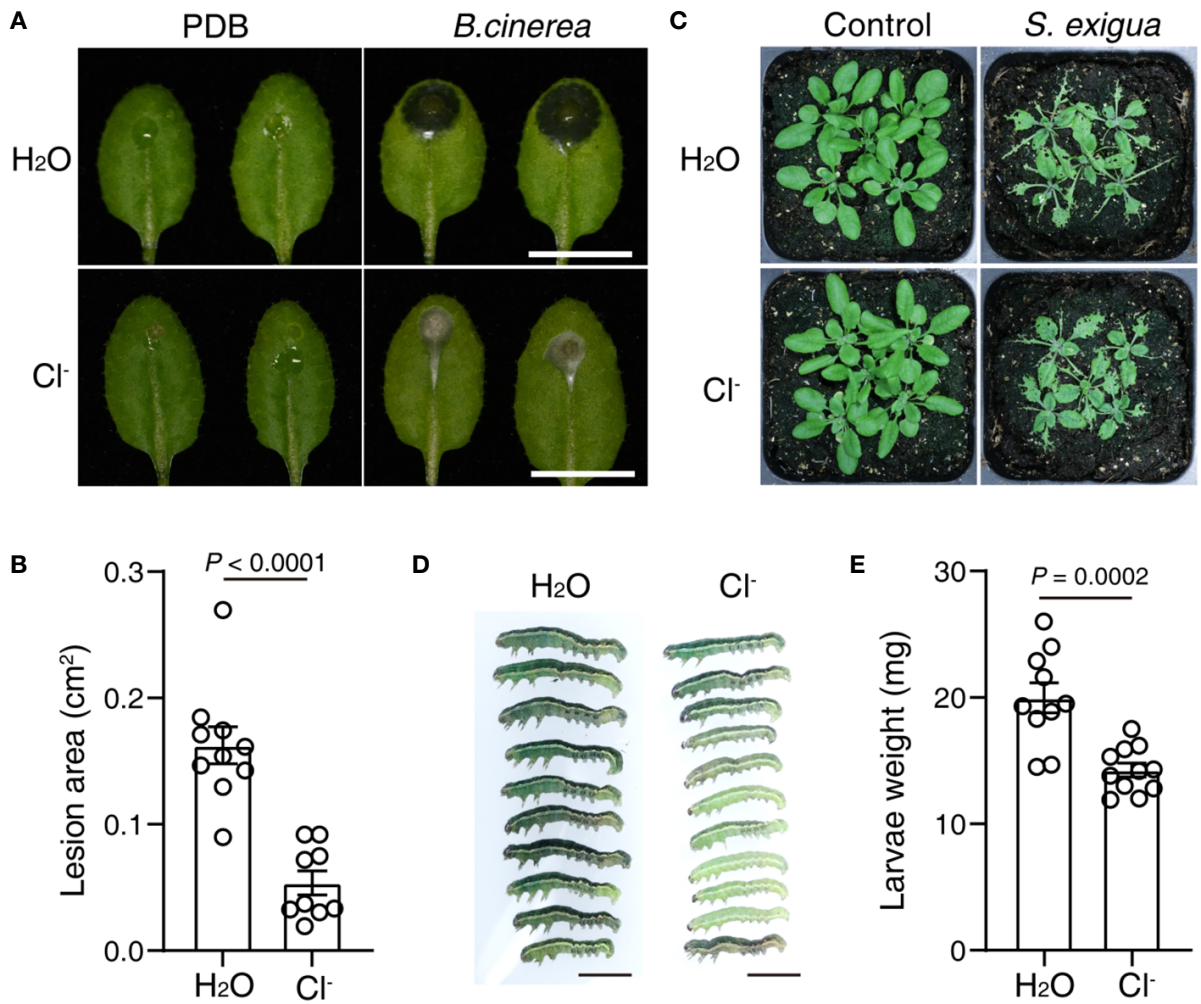
Cl^-^ treatment promotes plant resistance to *B*. *cinerea* and an herbivorous insect. Three-week-old soil-grown plants were irrigated with H_2_O or 300 mM Cl^-^. 24 h later, the leaves were drop-inoculated with 5 μL Mock (PDB), or 5 μL 1×10^5^ suspension of *B*. *cinerea* spores. **(A)** Photographs of representative leaves recorded 2 days post mock or spore infection, bar = 1 cm. **(B)** The quantification of lesion area in (**A**, n≥ 9). At 24 h after Cl^-^ treatment, five plants were fed with or without five second instar larvae of *S. exigua*. **(C)** Representative photos of plants taken after 4 days of larvae feeding, bar = 5 mm. **(D)** The growth of larvae after 4 days of feeding, bar = 5 mm. **(E)** Quantification of the larvae weight. Statistical differences were analyzed by Student’s *t*-test. Data are presented as mean values ± SEM. These experiments were repeated twice using independent samples.

Next, we investigated whether Cl^-^ irrigation could enhance plant resistance to a necrotrophic pathogen, the fungi *Botrytis cinerea*. At 24 h after Cl^-^ irrigation, a suspension of *B. cinerea* spores was dropped onto the leaves. As shown in **Figures 3A, B**, Cl^-^ irrigation greatly suppressed the lesions induced by *B. cinerea* infection. These results show that Cl^-^ irrigation can efficiently enhance plant resistance to necrotrophic pathogen infection.

We also tested that if Cl^-^ treatment could change plant resistance to herbivorous insect attack. At 24 h after Cl^-^ irrigation, second-instar larvae of *Spodoptera exigua* were placed on WT plants. Intriguingly, larval growth was greatly stunted in WT plants irrigated with Cl^-^ (**Figures 3C, D**). These results indicated that Cl^-^ treatment also enhanced plant resistance to an herbivorous insect.

## Discussion

The crosstalk between abiotic and biotic stress responses is complicated and far from being understood. In the present study, our collective data show that an abiotic treatment, irrigation with a Cl^-^ salt solution (MgCl_2_, KCl, and CaCl_2_), can remarkably enhance plant resistance to biotic stress, such as pathogen infection and herbivore attack. Furthermore, the enhanced plant resistance to a hemibiotrophic pathogen is only partially dependent on the SA pathway. We also demonstrated that Cl^-^ is the major ion suppressing the accelerated cell death in the *acd5* mutant.

### Cl^-^ salt suppresses cell death in *acd5*

As the main ions of salinity, Na^+^ and Cl^-^ have been long considered toxic ions that impair plant growth and development in the field (Munns and Tester, 2008; Tavakkoli et al., 2011). However, exploration of the crosstalk between abiotic and biotic stresses reveals that certain abiotic stresses or inorganic ions can improve plant growth, nutrient utilization, and plant responses to biotic stresses (Wang et al., 2013; Singh et al., 2014; Geilfus et al., 2015; Wege et al., 2017; Zhang et al., 2017; Franco-Navarro et al., 2019; Yang et al., 2019; Rosales et al., 2020). Cl^-^ salt treatment can change the morphology of leaf cells and reduce stomatal density, thus increasing water use efficiency, and improve photosynthetic performance, possibly by stimulating chloroplast biogenesis (Franco-Navarro et al., 2019). Cl^-^ also facilitates the utilization of NO_3_^-^ in plants, which provides a new guideline for developing agricultural fertilizers with an optimal NO_3_^-^/Cl^-^ ratio to reduce excessive NO_3_^-^ use (Rosales et al., 2020). Here, we discovered that Cl^-^ can also suppress the accelerated cell death in *acd5* (**Supplementary Figure S1**). This indicates that, besides promoting plant growth and nitrogen use, Cl^-^ also participates in cell death regulation in plants, possibly by provoking antagonistic crosstalk between the phytohormone abscisic acid and the SA pathway, as observed for NaCl treatment (Yang et al., 2019). Since Cl^-^ channels and Cl^-^ itself as a signaling ion are tightly related to cell proliferation and innate immunity in animal cells (Valdivieso and Santa-Coloma, 2019; Lüscher et al., 2020; Chen et al., 2022), we also suspect that Cl^-^ may inhibit cell death in *acd5* in a phytohormone-independent manner. Therefore, further research to decipher the molecular mechanism, for example by screening for suppressors of Cl^–^mediated suppression of cell death in *acd5*, should provide interesting insight.

### Cl^-^ as a beneficial ion enhances plant resistance to biotic stresses

Compatible or incompatible plant–microbe interactions show differing extents of host cell death. We found that Cl^-^ salt treatment effectively suppressed the cell death caused by not only the hemibiotrophic pathogen DG3, which undergoes a compatible interaction with Arabidopsis, but also DG6, in which recognition of the effector protein avrRpt2 by plant defenses induces an incompatible interaction (**Figure 1**; Yao and Greenberg, 2006). Cl^-^ also enhanced plant resistance to pathogen infection by suppressing bacterial multiplication in intercellular spaces, and this enhancement is partially dependent on the SA pathway but not PTI responses, especially those mediated by FLS2 (**Figure 2**). This result indicated that the mechanism by which Cl^-^ treatment enhanced disease resistance differed from that resulting from anion channel mutants or stimulated by channel inhibitors (Guo et al., 2014a, 2014b). Moreover, in the absence the pathogen infection, the genes related to immune responses and defense metabolites were downregulated (**Supplementary Figure S4D**), which is consistent with the observation that salt treatment suppresses the SA pathway. In addition, this inhibitory effect ceased when plants encountered a pathogen infection. These data suggested that the effect of chloride salt on biotic stress-responsive genes can also be regulated by upstream signaling pathways that are triggered by other environmental clues. RNA sequencing data showed the genes related to responses to toxic substance and benzene-containing compound metabolic process were enhanced in Cl^–^DG3 compared with H_2_O-DG3 treatment (**Supplementary Figure S4**), indicating these two processes play a role in Cl^-^ salt–induced plant biotic resistance response, although the molecular mechanism needs further elucidation.

Cl^-^ treatment also protected plants from the necrotrophic fungi *B. cinerea* and the herbivore *S. exigua*, which involve jasmonic acid (JA) signaling (**Figure 3**). This broad-spectrum resistance and the observation that the SA and JA pathways undergo antagonistic crosstalk (Pieterse et al., 2012), suggest that Cl^-^ treatment does not merely exploit phytohormone pathways to enhance plant biotic resistance. We suspect Cl^-^ could stimulate the accumulation of defense metabolites that are toxic to necrotrophic fungi and insects, a hypothesis that will need further validation.

Screening for suppressors of Cl^–^suppressed cell death in *acd5* may enable us to uncover the effect of Cl^-^ on plant cell death, and we will also learn from experiments in mammalian and yeast systems. Identifying the defense metabolites that are differentially abundant in plants under the combined treatment of Cl^-^ irrigation and biotic stress may also be helpful and intriguing. Taken together, this research provides new clues to study the crosstalk between abiotic and biotic responses and underscores the influences and biological functions of the inorganic ion Cl^-^ on plant cell death and defensive mechanisms.

## Data availability statement

The datasets presented in this study can be found in online repositories. The names of the repository/repositories and accession number(s) can be found below: BioProject, PRJNA1073023.

## Author contributions

YY: Conceptualization, Data curation, Methodology, Writing – original draft, Formal analysis, Funding acquisition. CY: Data curation, Methodology, Writing – original draft. JZ: Methodology, Writing – original draft. LX: Supervision, Writing – original draft. NY: Conceptualization, Funding acquisition, Supervision, Writing – review & editing, Resources.

## Supplementary material

Supplementary Figure S1 Salts containing chloride ions suppress the cell death phenotype in *acd5* plants. **(A)** Three-week-old soil-grown plants were irrigated with H_2_O or 300 mM NaCl, 300 mM KCl, 300 mM NaNO_3_, 300 mM KNO_3_, 150 mM Na_2_SO_4_, 150 mM K_2_SO_4_, or 150 mM CaCl_2_ once to soil capacity. The phenotypes were recorded 2 weeks later, bar = 1 cm, inner bar = 2 mm. This experiment was repeated twice with similar results using independent samples (n= 12). **(B)** Cl^-^ pretreatment suppresses the cell death phenotype of the *acd5* mutant. Three-week-old soil-grown plants were irrigated with H_2_O or 300 mM Cl^-^ (60 mM KCl, 60 mM MgCl_2_, 60 mM CaCl_2_) once to soil capacity. The phenotypes were recorded 2 weeks later, bar = 1 cm. This experiment was repeated at least twice with similar results using independent samples (n= 12).

Supplementary Figure S2 Cl^-^ but not the cations is vital for the cell death suppression. Three-week-old soil-grown plants were irrigated with H_2_O, 300 mM Cl^-^, or a salt mixture (60 mM KNO_3_, 60 mM MgSO_4_, and 60 mM Ca(NO_3_)_2_). 24 h later, the 3^rd^ and the 4^th^ leaf were infiltrated with Mock (10 mM MgSO_4_), or *Pma* DG3 (OD = 0.001). **(A)** Representative leaves recorded 2 dpi with Mock or *Pma* DG3 infiltration (bar = 5 mm). **(B)** Representative pictures of cell death visualized by trypan blue straining. Samples were detached and stained at the indicated time points, bar = 1 mm. **(C)** Statistical analysis of cell-death lesions in (**B**, n ≥ 12). The relative cell death lesion area per leaf was quantified as a percentage of lesion area in leaf area photographed under a stereomicroscope and measured by Image J software. Data are presented as mean values ± SEM. Statistical differences were analyzed by ANOVA *post hoc* tests (*P* < 0.05) and significantly different samples are indicated with different letters. This experiment was repeated twice using independent samples.

Supplementary Figure S3 Spray treatment with Cl^-^ enhances plant resistance to DG3. Three-week-old soil-grown plants were sprayed with H_2_O, 100 mM, 200 mM, or 300 mM Cl^-^ containing 0.02% Silwet-77. At 24 h later, the plants were sprayed with Mock (10 mM MgSO_4_ and 0.02% Silwet-77) treatment or *Pseudomonas syringae* pv *maculicola* (*Pma*) DG3 (OD = 0.05, 0.02% Silwet-77). Five plants were tested for each treatment. **(A)** Representative plant showing cell death visualized by trypan blue staining. Samples were harvested 2 days post DG3 spraying, bar = 1 cm. **(B)** The representative cell death phenotype of the 3^rd^ and 4^th^ leaves detached from plants in **(A)**, bar = 5 mm. **(C)** The cell death lesions recorded by stereo microscopy, bar = 1 mm. **(D)** Statistical analysis of cell death lesions in **(C)**. The cell death area per leaf was quantified as the ratio of lesion area under a stereomicroscope, and analyzed using Image J software. Data are presented as mean values ± SEM. Statistical differences were analyzed by ANOVA *post hoc* tests (*P* < 0.05) and significantly different samples are indicated with different letters. This experiment was repeated twice with similar results using independent samples.

Supplementary Figure S4 Transcriptome analysis upon treatment with Cl^-^ and DG3. Three-week-old soil-grown plants were irrigated with H_2_O or 300 mM Cl^-^. 24 h later, the 3^rd^ and the 4^th^ leaf were infiltrated with Mock (10 mM MgSO_4_) treatment, or DG3 (OD = 0.001). Samples were collected 9 h post DG3 infection for total RNA isolation and sequencing. Data analysis was based on Log FC ≥ 1 and *P* < 0.05. **(A, C)** Venn diagrams showing the numbers of genes upregulated **(A)** or downregulated **(C)** upon combined treatment with salt and infection with DG3. **(B, D)** Gene ontology and cluster analysis for the biological processes upregulated **(B)** or downregulated **(D)** upon treatment with salt and infection with DG3.

## References

[B1] Bi F. C. Liu Z. Wu J. X. Liang H. Xi X. L. Fang C.. (2014). Loss of ceramide kinase in Arabidopsis impairs defenses and promotes ceramide accumulation and mitochondrial H_2_O_2_ bursts. Plant Cell 26, 3449–3467. doi: 10.1105/tpc.114.127050 25149397 PMC4176443

[B2] Chen L. Guan W. J. Qiu Z. E. Xu J. B. Bai X. Hou X. C.. (2022). SARS-CoV-2 nucleocapsid protein triggers hyperinflammation via protein-protein interaction-mediated intracellular Cl^-^ accumulation in respiratory epithelium. Signal Transduction Targeted Ther. 7, 255. doi: 10.1038/s41392-022-01048-1 PMC9328007 35896532

[B3] Davenport R. J. Munoz-Mayor A. Jha D. Essah P. A. Rus A. Tester M. (2007). The Na^+^ transporter AtHKT1;1 controls retrieval of Na^+^ from the xylem in Arabidopsis. Plant Cell Environ. 30, 497–507. doi: 10.1111/j.1365-3040.2007.01637.x 17324235

[B4] Franco-Navarro J. D. Rosales M. A. Cubero-Font P. Calvo P. Alvarez R. Diaz-Espejo A.. (2019). Chloride as a macronutrient increases water-use efficiency by anatomically driven reduced stomatal conductance and increased mesophyll diffusion to CO_2_ . Plant J. 99, 815–831. doi: 10.1111/tpj.14423 31148340

[B5] Geilfus C. M. Mithofer A. Ludwig-Muller J. Zorb C. Muehling K. H. (2015). Chloride- inducible transient apoplastic alkalinizations induce stomata closure by controlling abscisic acid distribution between leaf apoplast and guard cells in salt-stressed *Vicia faba* . New Phytol. 208, 803–816. doi: 10.1111/nph.13507 26096890

[B6] Guo W. Wang C. Zuo Z. Qiu J. L. (2014a). The roles of anion channels in Arabidopsis immunity. Plant Signaling Behav. 9, e29230. doi: 10.4161/psb.29230 PMC4203573 25763497

[B7] Guo W. Zuo Z. Cheng X. Sun J. Li H. Li L.. (2014b). The chloride channel family gene CLCd negatively regulates pathogen-associated molecular pattern (PAMP)-triggered immunity in Arabidopsis. J. Exp. Bot. 65, 1205–1215. doi: 10.1093/jxb/ert484 24449384 PMC3935575

[B8] Hasegawa P. M. Bressan R. A. Zhu J. K. Bohnert H. J. (2000). Plant cellular and molecular responses to high salinity. Annu. Rev. Plant Physiol. Plant Mol. Biol. 51, 463–499. doi: 10.1146/annurev.arplant.51.1.463 15012199

[B9] Huang L. Q. Chen D. K. Li P. P. Bao H. N. Liu H. Z. Yin J.. (2021). Jasmonates modulate sphingolipid metabolism and accelerate cell death in the ceramide kinase mutant *acd5* . Plant Physiol. 187, 1713–1727. doi: 10.1093/plphys/kiab362 34618068 PMC8566286

[B10] Kong X. Zhang C. Zheng H. Sun M. Zhang F. Zhang M.. (2020). Antagonistic interaction between Auxin and SA signaling pathways regulates bacterial infection through lateral root in Arabidopsis. Cell Rep. 32, 108060. doi: 10.1016/j.celrep.2020.108060 32846118

[B11] Liang H. Yao N. Song J. T. Luo S. Lu H. Greenberg J. T. (2003). Ceramides modulate programmed cell death in plants. Genes Dev. 17, 2636–2641. doi: 10.1101/gad.1140503 14563678 PMC280613

[B12] Livak K. J. Schmittgen T. D. (2001). Analysis of relative gene expression data using real-time quantitative PCR and the 2(^-△△CT^) method. Methods 25, 402–408. doi: 10.1006/meth.2001.1262 11846609

[B13] Lüscher B. P. Vachel L. Ohana E. Muallem S. (2020). Cl^-^ as a bona fide signaling ion. Am. J. Physiol. Cell Physiol. 318, C125–C136. doi: 10.1152/ajpcell.00354.2019 31693396 PMC6985830

[B14] Munns R. Tester M. (2008). Mechanisms of salinity tolerance. Annu. Rev. Plant Physiol. Plant Mol. Biol. 59, 651–681. doi: 10.1146/annurev.arplant.59.032607.092911 18444910

[B15] Pieterse C. M. van der Does D. Zamioudis C. Leon-Reyes A. Van Wees S. C. (2012). Hormonal modulation of plant immunity. Annu. Rev. Cell Dev. Biol. 28, 489–521. doi: 10.1146/annurev-cellbio-092910-154055 22559264

[B16] Rosales M. A. Franco-Navarro J. D. Peinado-Torrubia P. Díaz-Rueda P. Alvarez R. Colmenero-Flores J. M. (2020). Chloride improves nitrate utilization and NUE in plants. Front. Plant Sci. 11, 442. doi: 10.3389/fpls.2020.00442 32528483 PMC7264407

[B17] Singh P. Yekondi S. Chen P. W. Tsai C. H. Yu C. W. Wu K.. (2014). Environmental history modulates Arabidopsis pattern-triggered immunity in a HISTONE ACETYLTRANSFERASE1-dependent manner. Plant Cell 26, 2676–2688. doi: 10.1105/tpc.114.123356 24963055 PMC4114959

[B18] Sirén J. Välimäki N. Mäkinen V. (2014). Indexing graphs for path queries with applications in genome research. IEEE/ACM Trans. Comput. Biol. Bioinform. 11, 375–388. doi: 10.1109/Tcbb.2013.2297101 26355784

[B19] Tavakkoli E. Fatehi F. Coventry S. Rengasamy P. McDonald G. K. (2011). Additive effects of Na^+^ and Cl^-^ ions on barley growth under salinity stress. J. Exp. Bot. 62, 2189–2203. doi: 10.1093/jxb/erq422 21273334 PMC3060698

[B20] Valdivieso A. G. Santa-Coloma T. A. (2019). The chloride anion as a signalling effector. Biol. Rev. 94, 1839–1856. doi: 10.1111/brv.12536 31231963

[B21] Wang L. K. Feng Z. X. Wang X. Wang X. W. Zhang X. G. (2010). DEGseq: an R package for identifying differentially expressed genes from RNA-seq data. Bioinformatics 26, 136–138. doi: 10.1093/bioinformatics/btp612 19855105

[B22] Wang Y. Chen Z. H. Zhang B. Hills A. Blatt M. R. (2013). PYR/PYL/RCAR abscisic acid receptors regulate K^+^ and Cl^-^ channels through reactive oxygen species- mediated activation of Ca2+ channels at the plasma membrane of intact *Arabidopsis* guard cells. Plant Physiol. 163, 566–577. doi: 10.1104/pp.113.219758 23899646 PMC3793038

[B23] Wege S. Gilliham M. Henderson S. W. (2017). Chloride: not simply a ‘cheap osmoticum’, but a beneficial plant macronutrient. J. Exp. Bot. 68, 3057–3069. doi: 10.1093/jxb/erx050 28379459

[B24] Yang Y. B. Yin J. Huang L. Q. Li J. Chen D. K. Yao N. (2019). Salt enhances disease resistance and suppresses cell death in ceramide kinase mutants. Plant Physiol. 181, 319–331. doi: 10.1104/pp.19.00613 31243063 PMC6716259

[B25] Yao N. Greenberg J. T. (2006). Arabidopsis ACCELERATED CELL DEATH2 modulates programmed cell death. Plant Cell 18, 397–411. doi: 10.1105/tpc.105.036251 16387834 PMC1356547

[B26] Yu G. C. Wang L. G. Han Y. Y. He Q. Y. (2012). clusterProfiler: an R Package for comparing biological themes among gene clusters. Omics. 16, 284–287. doi: 10.1089/omi.2011.0118 22455463 PMC3339379

[B27] Zeng H. Y. Liu Y. Chen D. K. Bao H. N. Huang L. Q. Yin J.. (2021). The immune components ENHANCED DISEASE SUSCEPTIBILITY 1 and PHYTOALEXIN DEFICIENT 4 are required for cell death caused by overaccumulation of ceramides in Arabidopsis. Plant J. 107, 1447–1465. doi: 10.1111/tpj.15393 34180563

[B28] Zhang H. Zhao F. G. Tang R. J. Yu Y. Song J. Wang Y. (2017). Two tonoplast MATE proteins function as turgor-regulating chloride channels in *Arabidopsis* . Proc. Natl. Acad. Sci. United States America 114, E2036–E2045. doi: 10.1073/pnas.1616203114 PMC5347570 28202726

[B29] Zhang W. W. Yang H. Q. You S. Z. Fan S. L. Ran K. (2015a). *MhNCED3*, a gene encoding 9-cis-epoxycarotenoid dioxygenase in *Malus hupehensis* Rehd., enhances plant tolerance to Cl^-^ stress by reducing Cl^-^ accumulation. Plant Physiol. Biochem. 89, 85–91. doi: 10.1016/j.plaphy.2015.02.012 25725410

[B30] Zhang Y. Jin X. Ouyang Z. Li X. Liu B. Huang L.. (2015b). Vitamin B6 contributes to disease resistance against *Pseudomonas syringae* pv. tomato DC3000 and *Botrytis cinerea* in *Arabidopsis thaliana* . J. Plant Physiol. 175, 21–25. doi: 10.1016/j.jplph.2014.06.023 25460872

[B31] Zhu J. K. (2016). Abiotic stress signaling and responses in plants. Cell 167, 313–324. doi: 10.1016/j.cell.2016.08.029 27716505 PMC5104190

