# Supplementary material for: Chloride salt enhances plant resistance to biotic stresses
Source: Front Plant Sci. 2024 Jun 4;15:1385164. doi: 10.3389/fpls.2024.1385164 (PMC11183330; doi:10.3389/fpls.2024.1385164)
Supplement: Supplementary Figure S1 — Salts containing chloride ions suppress the cell death phenotype in acd5 plants. (A) Three-week-old soil-grown plants were irrigated with H2O or 300 mM NaCl, 300 mM KCl, 300 mM NaNO3, 300 mM KNO3, 150 mM Na2SO4, 150 mM K2SO4, or 150 mM CaCl2 once to soil capacity. The phenotypes were recorded 2 weeks later, bar = 1 cm, inner bar = 2 mm. This experiment was repeated twice with similar results using independent samples (n= 12). (B) Cl- pretreatment suppresses the cell death phenotype of the acd5 mutant. Three-week-old soil-grown plants were irrigated with H2O or 300 mM Cl- (60 mM KCl, 60 mM MgCl2, 60 mM CaCl2) once to soil capacity. The phenotypes were recorded 2 weeks later, bar = 1 cm. This experiment was repeated at least twice with similar results using independent samples (n= 12). [file DataSheet_1.docx]

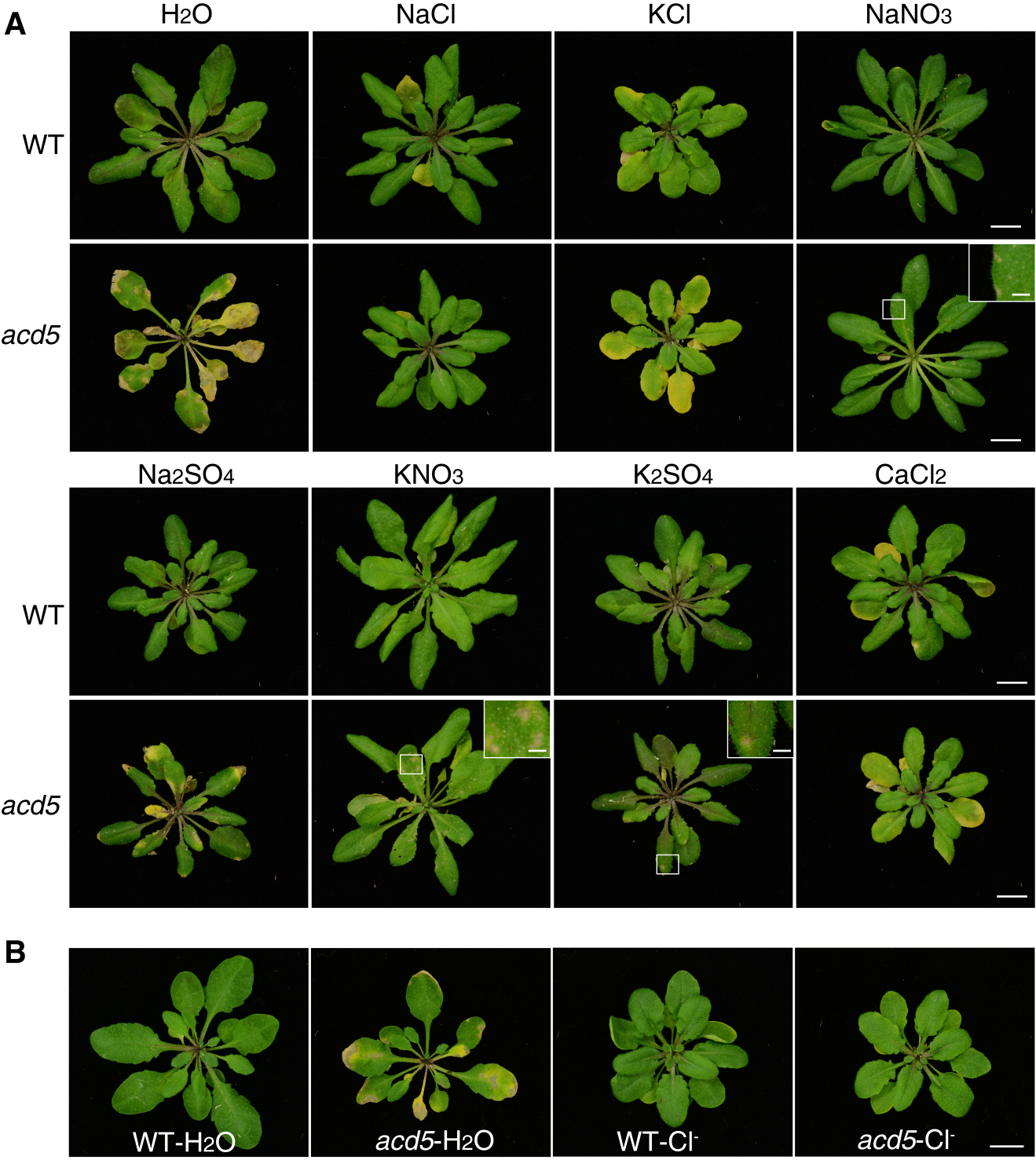


**Figure S1** Salts containing chloride ions suppress the cell death phenotype in *acd5* plants

(**A**) Three-week-old soil-grown plants were irrigated with H_2_O or 300 mM NaCl, 300 mM KCl, 300 mM NaNO_3_, 300 mM KNO_3_, 150 mM Na_2_SO_4_, 150 mM K_2_SO_4_, or 150 mM CaCl_2_ once to soil capacity. The phenotypes were recorded 2 weeks later, bar = 1 cm, inner bar = 2 mm. This experiment was repeated twice with similar results using independent samples (n= 12).

(**B**) Cl^-^ pretreatment suppresses the cell death phenotype of the *acd5* mutant. Three-week-old soil-grown plants were irrigated with H_2_O or 300 mM Cl^-^ (60 mM KCl, 60 mM MgCl_2_, 60 mM CaCl_2_) once to soil capacity. The phenotypes were recorded 2 weeks later, bar = 1 cm. This experiment was repeated at least twice with similar results using independent samples (n= 12).

**
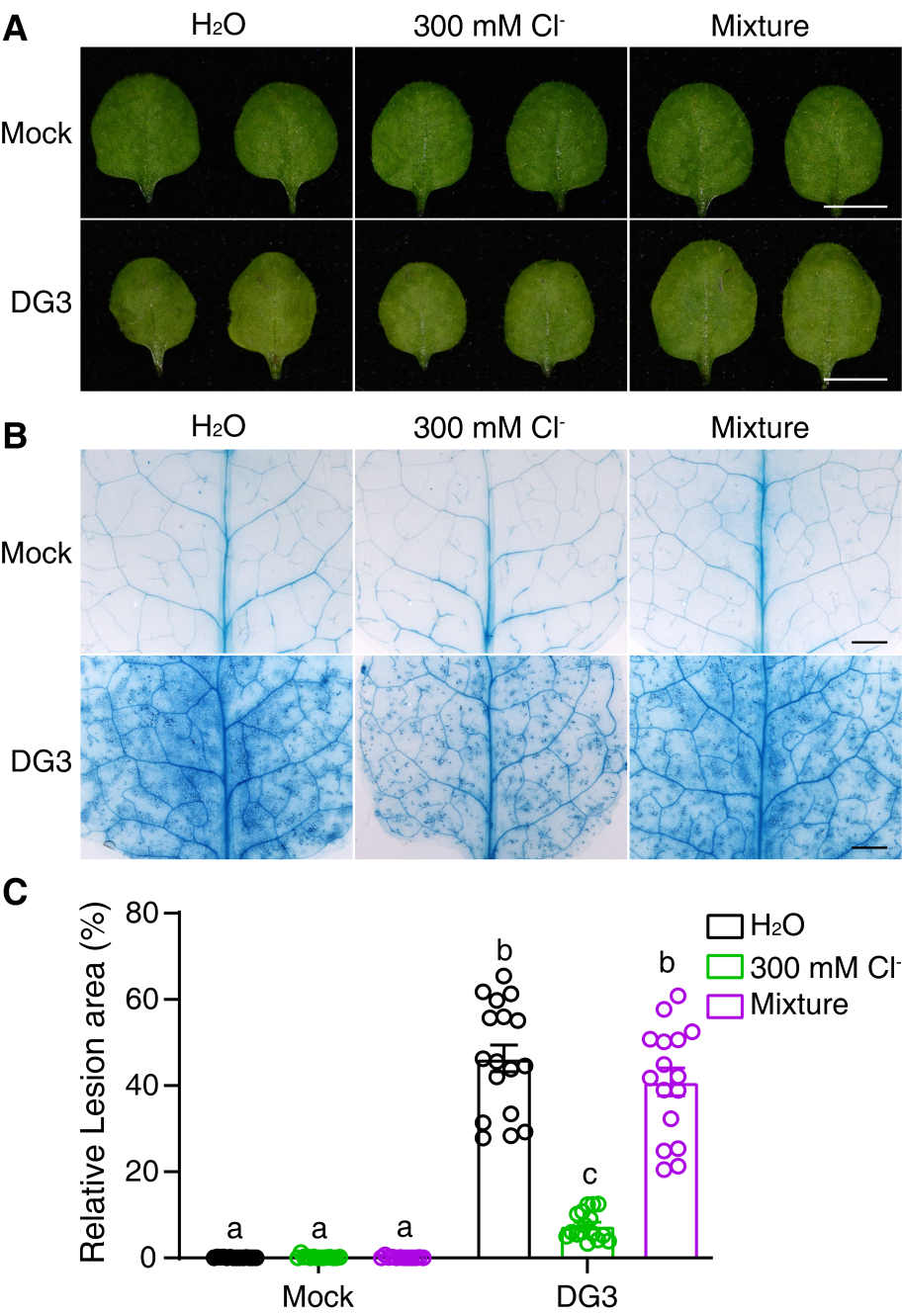
**

**Figure S2** Cl^-^ but not the cations is vital for the cell death suppression

Three-week-old soil-grown plants were irrigated with H_2_O, 300 mM Cl^-^, or a salt mixture (60 mM KNO_3_, 60 mM MgSO_4_, and 60 mM Ca(NO_3_)_2_). 24 h later, the 3^rd^ and the 4^th^ leaf were infiltrated with Mock (10 mM MgSO_4_), or *Pma* DG3 (OD = 0.001). **A**. Representative leaves recorded 2 dpi with Mock or *Pma* DG3 infiltration (bar = 5 mm). **B.** Representative pictures of cell death visualized by trypan blue straining. Samples were detached and stained at the indicated time points, bar = 1 mm. **C**. Statistical analysis of cell-death lesions in (**B**, n ≥ 12). The relative cell death lesion area per leaf was quantified as a percentage of lesion area in leaf area photographed under a stereomicroscope and measured by Image J software. Data are presented as mean values ± SEM. Statistical differences were analyzed by ANOVA post hoc tests (*P* < 0.05) and significantly different samples are indicated with different letters. This experiment was repeated twice using independent samples.

**
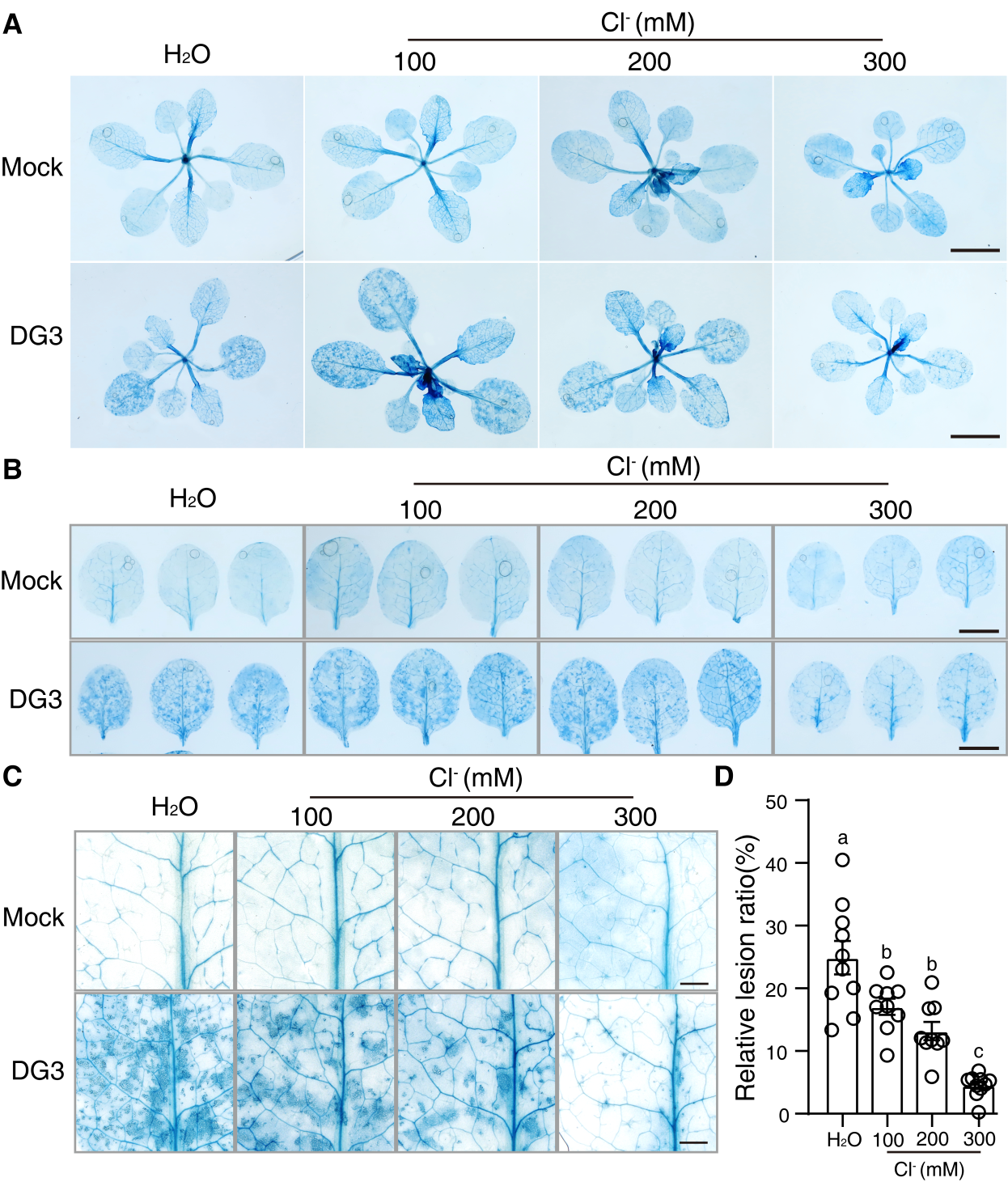
**

**Figure S3** Spray treatment with Cl^-^ enhances plant resistance to DG3

Three-week-old soil-grown plants were sprayed with H_2_O, 100 mM, 200 mM, or 300 mM Cl^-^ containing 0.02% Silwet-77. At 24 h later, the plants were sprayed with Mock (10 mM MgSO_4_ and 0.02% Silwet-77) treatment or *Pseudomonas syringae* pv *maculicola* (*Pma*) DG3 (OD = 0.05, 0.02% Silwet-77). Five plants were tested for each treatment. **A.** Representative plant showing cell death visualized by trypan blue staining. Samples were harvested 2 days post DG3 spraying, bar = 1 cm. **B**. The representative cell death phenotype of the 3^rd^ and 4^th^ leaves detached from plants in (**A**), bar = 5 mm. **C**. The cell death lesions recorded by stereo microscopy, bar = 1 mm. **D**. Statistical analysis of cell death lesions in (**C**). The cell death area per leaf was quantified as the ratio of lesion area under a stereomicroscope, and analyzed using Image J software. Data are presented as mean values ± SEM. Statistical differences were analyzed by ANOVA post hoc tests (*P* < 0.05) and significantly different samples are indicated with different letters. This experiment was repeated twice with similar results using independent samples.


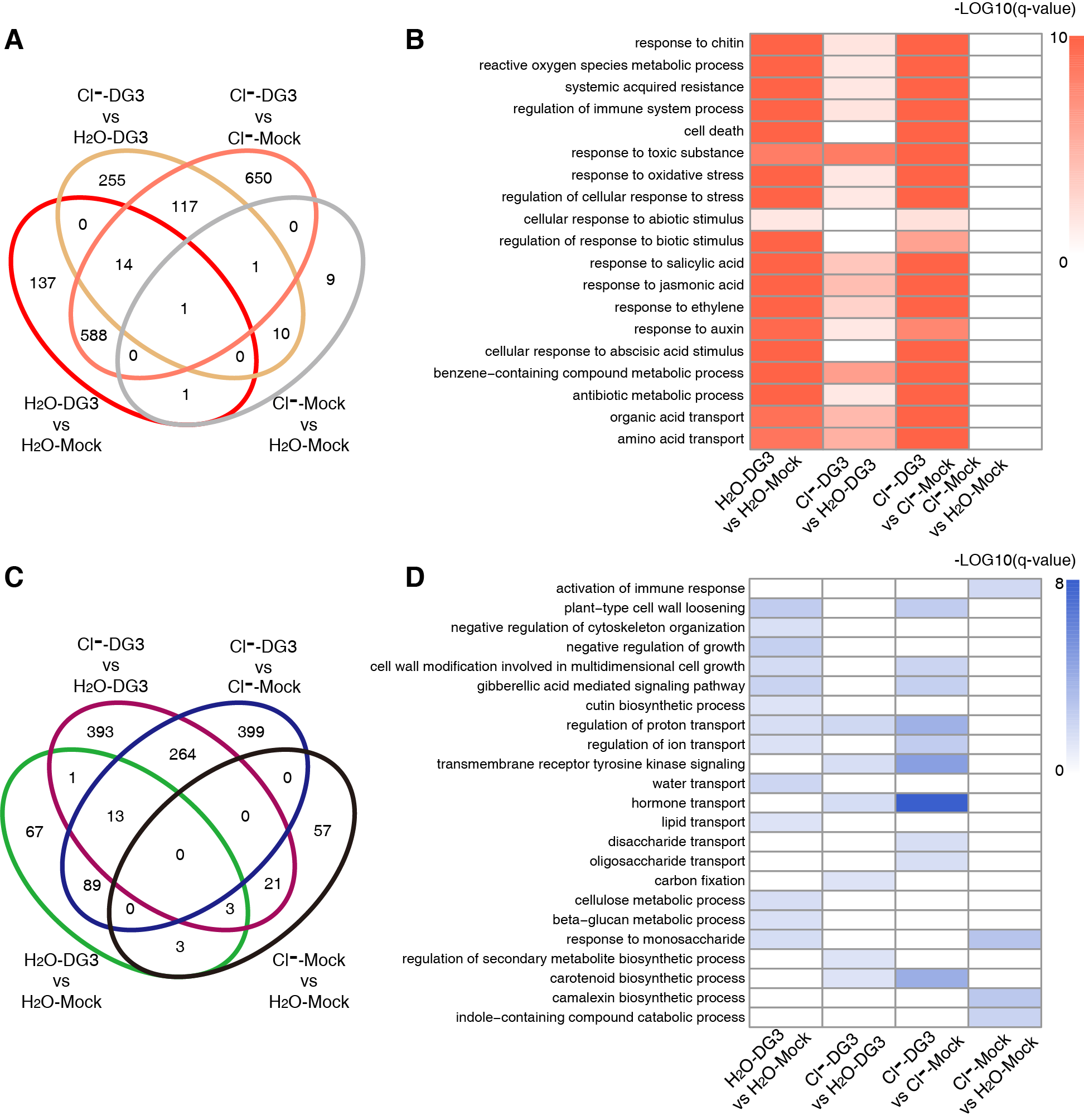


**Figure S4** Transcriptome analysis upon treatment with Cl^-^ and DG3

Three-week-old soil-grown plants were irrigated with H_2_O or 300 mM Cl^-^. 24 h later, the 3^rd^ and the 4^th^ leaf were infiltrated with Mock (10 mM MgSO_4_) treatment, or DG3 (OD = 0.001). Samples were collected 9 h post DG3 infection for total RNA isolation and sequencing. Data analysis was based on Log FC ≥ 1 and *P* < 0.05. **A**, **C**. Venn diagrams showing the numbers of genes upregulated (**A**) or downregulated (**C**) upon combined treatment with salt and infection with DG3. **B**, **D**. Gene ontology and cluster analysis for the biological processes upregulated (**B**) or downregulated (**D**) upon treatment with salt and infection with DG3.
